# Temporal Comparison of Virulence-Associated Characteristics and Metabolic Activity in Fluoroquinolone-Resistant *Escherichia coli* O25 ST131 Urinary Isolates from Croatia

**DOI:** 10.3390/pathogens15101020

**Published:** 2026-09-28

**Authors:** Jasmina Vraneš, Maja Anušić, Vladimira Tičić, Sandra Šuto, Ivana Kolčić, Tatjana Marijan, Branka Bedenić

**Affiliations:** 1 Department of Clinical Microbiology, Dr Andrija Štampar Teaching Institute of Public Health, 10000 Zagreb, Croatia; maja.anusic@stampar.hr (M.A.); vladimira.ticic@stampar.hr (V.T.); sandra.suto@stampar.hr (S.Š.); tatjana.marijan@stampar.hr (T.M.); 2 Department of Medical Microbiology and Parasitology, University of Zagreb School of Medicine, 10000 Zagreb, Croatia; branka.bedenic@kbc-zagreb.hr; 3 Department of Public Health, Dr Andrija Štampar Teaching Institute of Public Health, 10000 Zagreb, Croatia; ivana.kolcic@stampar.hr; 4 Department of Public Health, University of Split School of Medicine, 21000 Split, Croatia; 5 Clinical Department for Clinical Microbiology, Infection Control and Prevention, University Hospital Center Zagreb, 10000 Zagreb, Croatia

**Keywords:** bacterial fitness, bacterial virulence, clonal spreading, molecular epidemiology, multidrug-resistant *E. coli*

## Abstract

The aim of this study was to compare the virulence-associated characteristics and metabolic activity of fluoroquinolone-resistant (FR) *Escherichia coli* O25 ST131 isolates recovered from urine samples of outpatients in Zagreb, Croatia, during two study periods, 2011/2012 and 2017/2018. The O25 ST131 clonal lineage was identified by rapid multiplex PCR targeting the *O25pabB* and *trpA* genes. PCR was used to detect *bla*CTX-M, *bla*CTX-M-15, *bla*TEM, and *bla*SHV β-lactamase genes, as well as the virulence-associated genes *fimH*, *papC*, *hlyA*, *cnf1*, and *traT.* Metabolic activity was evaluated using the VITEK 2 GN card, and serum resistance was assessed using a serum susceptibility assay. Differences between study periods were analyzed using linear regression and the chi-square test. A total of 4354 and 7717 *E. coli* isolates were recovered during the first and second study periods, respectively, and 170 FR *E. coli* O25 isolates were collected. Among the O25 ST131 isolates, 30/45 (66.7%) from the first study period and 75/114 (65.8%) from the second study period were ESBL-positive, with CTX-M-15 predominating. The *fimH* gene was detected in all 159 O25 ST131 isolates (100%), whereas *hlyA*, *papC*, *cnf1*, and *traT* were detected in 35 (22%), 43 (27%), 35 (22%), and 123 (77%) isolates, respectively. Compared with the first study period, isolates from the second period showed significantly higher frequency of *papC*, *hlyA*, and *cnf1*, as well as higher serum resistance and metabolic potential (*p* < 0.01). These findings demonstrate temporal differences in selected virulence-associated characteristics, serum resistance, and metabolic activity among FR *E. coli* O25 ST131 isolates and support the need for continued surveillance of this clinically important clonal lineage in the community.

## 1. Introduction

Antimicrobial resistance (AMR) is a major global public health challenge, with substantial consequences for the prevention and treatment of infectious diseases in humans and animals. Antibiotics remain essential for modern medicine and veterinary practice, and the increasing prevalence of multidrug-resistant (MDR) bacteria threatens the effectiveness of established therapeutic strategies. MDR bacteria are now widespread globally, with resistant organisms capable of spreading locally, nationally, and internationally through human and animal movement and travel. This contributes to increased morbidity and mortality and imposes a substantial economic burden on healthcare systems. Coordinated efforts are therefore required to preserve the effectiveness of antimicrobial agents for future generations [1].

*Escherichia coli* O25 ST131 is a globally disseminated MDR clonal lineage that is predominant among extraintestinal pathogenic *E. coli* (ExPEC). The ST131 lineage comprises several genetic clades (A, B, and C), with clade C being the most prevalent worldwide [2,3]. The sequential acquisition of antimicrobial resistance and virulence-associated determinants, including genes carried on mobile genetic elements, contributed to the emergence and global dissemination of clade C and its subclades C1 and C2 during the 1990s and 2000s [2]. Although ST131 has substantially influenced the population structure of ExPEC, the specific combination of characteristics underlying its successful global dissemination remains incompletely understood [2]. Reports of ST131 in humans, wild and companion animals, food-producing animals, and the environment have increased worldwide [4,5,6,7,8,9]. Although the potential transmission of MDR *E. coli* between animals and humans has received increasing attention, the relationships among humans, animals, the environment, and the transmission of antimicrobial-resistant *E. coli* remain incompletely understood.

Urinary tract infections (UTIs) are among the most common bacterial infections in humans and are a major indication for antimicrobial treatment. The global dissemination of MDR *E. coli* ST131 has created additional challenges for the treatment of community-acquired infections, including UTIs. The ability of ST131 to persistently colonize the intestinal tract, including in the absence of antimicrobial exposure, may contribute to its persistence and dissemination and indicates that antimicrobial stewardship alone may not be sufficient to control its spread [10]. Understanding the bacterial and host factors contributing to the successful dissemination of ST131 is therefore important for developing strategies to limit its expansion [10]. AMR of uropathogenic *E. coli* (UPEC) to first-line antimicrobial agents, including co-trimoxazole, ciprofloxacin, and third-generation cephalosporins, as well as to last-line agents such as carbapenems, is of particular concern [11]. The convergence of antimicrobial resistance and virulence-associated characteristics in successful *E. coli* lineages highlights the need for continued investigation of the determinants underlying their persistence, dissemination, and pathogenic potential in animals, humans and the environment.

Bacterial metabolic activity may contribute to fitness by influencing the ability of bacteria to obtain energy, synthesize cellular components, and adapt to specific ecological niches. Characterizing metabolic and virulence-associated properties of successful antimicrobial-resistant clones may therefore provide insight into their persistence and dissemination.

The aim of this study was to compare selected virulence-associated characteristics and metabolic activity of fluoroquinolone-resistant (FR) *E. coli* O25 ST131 isolates recovered from midstream urine samples collected during two study periods, 2011/2012 and 2017/2018, from outpatients with community-acquired urinary tract infections (CA-UTIs) in Zagreb, Croatia. In this study, CA-UTI refers to acute urinary tract infection in outpatients without a documented history of recent hospitalization who underwent microbiological investigation because of symptoms suggestive of acute UTI.

Data on the dissemination of the ST131 clone in Croatia remain limited. The clone was first detected in Croatia in 2009, although the corresponding study investigated hospital isolates collected in 2002/2003. In a previous study of MDR *E. coli* O25 ST131 in the Croatian outpatient population, antimicrobial resistance patterns were compared between isolates recovered from urine samples during 2011/2012 and 2017/2018 [12,13]. In the present study, we further investigated virulence-associated characteristics and metabolic activity of isolates from these two periods in relation to their antimicrobial resistance phenotype, with the aim of assessing temporal differences in characteristics potentially contributing to the persistence and dissemination of this clonal lineage.

## 2. Materials and Methods

### 2.1. Urine Culture and Bacterial Identification

Midstream urine samples were inoculated onto non-selective blood agar and differential selective MacConkey agar (Oxoid Ltd., Basingstoke, Hampshire, UK). *E. coli* was identified using standard biochemical tests, including glucose and lactose fermentation, indole production, citrate and phenylalanine utilization, and lysine and/or ornithine decarboxylation.

### 2.2. Bacterial Isolates

This retrospective cross-sectional study investigated non-duplicate, consecutive fluoroquinolone-resistant (FR) *E. coli* O25 ST131 isolates obtained from midstream urine samples received at the Dr. Andrija Štampar Teaching Institute of Public Health, Zagreb, Croatia, during two study periods: March 2011 to January 2012 and October 2017 to August 2018. During the first study period, 4354 *E. coli* isolates were recovered from 29,544 urine samples, whereas during the second period, 7717 *E. coli* isolates were recovered from 52,193 urine samples.

Inclusion criteria were fluoroquinolone resistance, a positive leukocyte esterase test, and a bacterial count of ≥10^5^ CFU/mL of urine in outpatients diagnosed with acute urinary tract infection.

A total of 170 O25 isolates were selected from 519 FR *E. coli* isolates identified during the two study periods. Isolates were stored at −80 °C in commercial cryopreservation medium (Microbank, Pro-Lab Diagnostics, Richmond Hill, ON, Canada) and were used for further analysis.

### 2.3. Detection of Clonal Lineage E. coli O25 ST131

All 519 collected FR *E. coli* isolates were initially tested by agglutination with *E. coli* O25 antiserum (Denka Seiken Co., Ltd., Chuo-ku, Tokyo, Japan) according to the manufacturer’s instructions. The ST131 clonal group was subsequently detected among the 159/170 O25-positive FR *E. coli* isolates using a rapid PCR method with the primers O25pabB.F (5′-TCCAGCAGGTGCTGGATCGT-3′), O25pabB.R (5′-GCGAAATTTTTCGCCGTACTGT-3′), trpA.F (5′-GCTACGAATCTCTGTTTGCC-3′), and trpA.R (5′-GCAACGCGGCCTGGCGGAAG-3′), as previously described by Clermont et al. [14].

Briefly, PCR was performed in a total volume of 20 μL containing 2 μL of 10× buffer (Invitrogen, Thermo Fisher Scientific, Waltham, MA, USA), 2 μL of O25pabB.F primer (20 pmol, Microsynth AG, Balgach, Switzerland), 2 μL of O25pabB.R primer (20 pmol, Microsynth AG), 1.2 μL of trpA.F primer (12 pmol, Microsynth AG), 1.2 μL of trpA.R primer (12 pmol, Microsynth AG), 8 μL of dNTPs (20 μM, Invitrogen, Thermo Fisher Scientific), 0.2 μL of *Taq* polymerase (1 U, Invitrogen, Carlsbad, CA, USA), 0.6 μL of MgCl_2_ (5 mM), and 2.8 μL of bacterial lysate.

The PCR conditions were as follows: initial denaturation at 94 °C for 4 min, followed by 30 cycles of denaturation at 94 °C for 5 s and annealing at 65 °C for 10 s, with a final extension at 72 °C for 5 min. PCR products were analyzed using the MCE-202 MultiNA microchip electrophoresis system for DNA/RNA analysis (Shimadzu Co., Kyoto, Japan). An *E. coli* O25 ST131 isolate previously confirmed by multilocus sequence typing (MLST) was used as the positive control, and *E. coli* ATCC 25922 was used as the negative control.

### 2.4. Metabolic Profiling of FR E. coli O25ST131

Metabolic profiling was performed using the VITEK 2 Compact automated system (bioMérieux, Marcy-l’Étoile, France). The GN identification card provided very good or excellent identification results for all FR *E. coli* O25 ST131 isolates.

The metabolic activity of the 159 detected FR *E. coli* O25 ST131 isolates was calculated as the ratio of the number of positive biochemical reactions to the total number of 47 biochemical reactions tested, as previously described by Gibreel et al. [15].

### 2.5. Detection of FR and ESBL Phenotype

The FR and ESBL phenotypes were detected according to the methods recommended by EUCAST [16]. All isolated *E. coli* strains were routinely screened with a 5 μg ciprofloxacin disk and fluoroquinolone resistance in 159 O25 ST131 isolates was confirmed by using the microdilution method with ciprofloxacin concentrations ranging from 0.06 to 128 μg/mL. 

The presence of the ESBL phenotype in the 170 *E. coli* O25 isolates was detected by using a combined disk test and a double-disk synergy test. For the combination disk test, disks containing cefotaxime, ceftazidime, ceftriaxone, and cefepime, alone and in combination with clavulanic acid, were used. An increase in the inhibition zone diameter of ≥5 mm in the presence of clavulanic acid was interpreted as evidence of ESBL production. For the double-disk synergy test, disks containing cefotaxime, ceftazidime, and cefepime were placed in proximity to a disk containing amoxicillin–clavulanic acid. Distortion or enhancement of the cephalosporin inhibition zone toward the clavulanic acid-containing disk was interpreted as a confirmatory result for ESBL production.

### 2.6. Detection of CTX-M, CTX-M15, TEM and SHV β-Lactamases

PCR was performed to detect the *bla*CTX-M, *bla*CTX-M-15, *bla*TEM, and *bla*SHV β-lactamase genes in 105 ESBL-positive *E. coli* O25 ST131 isolates. The primers used for detection of CTX-M, CTX-M-15, TEM, and SHV β-lactamases are listed in Appendix A, Table A1.

The PCR targeting *bla*CTX-M-15 amplified the entire coding sequence of the *bla*CTX-M-15 gene, enabling identification of this specific allelic variant without sequencing.

For CTX-M detection, the PCR mixture had a final volume of 20 μL and contained 10 μL of Takara EmeraldAmp PCR Master Mix, 1 μL of each primer (20 pmol; Microsynth AG), 7 μL of PCR-grade water, and 1 μL of bacterial lysate. The PCR conditions were as follows: initial denaturation at 95 °C for 5 min, followed by 35 cycles of 95 °C for 1 min, 53 °C for 30 s, and 72 °C for 50 s, with a final extension at 72 °C for 10 min [17].

The presence of the mobile insertion sequence IS26 upstream of *bla*CTX-M was determined by PCR mapping using a forward primer targeting the insertion element in combination with the universal reverse primer MA3 targeting the *bla*CTX-M gene as previously described [18]. 

For CTX-M-15 detection, the PCR mixture had a final volume of 20 μL and contained 10 μL of Takara EmeraldAmp PCR Master Mix, 1 μL of each primer (20 pmol; Microsynth AG), 7 μL of PCR-grade water, and 1 μL of bacterial lysate. The PCR conditions were as follows: initial denaturation at 94 °C for 3 min, followed by 35 cycles of 95 °C for 30 s, 55 °C for 30 s, and 72 °C for 45 s, with a final extension at 72 °C for 5 min [19].

For *bla*TEM detection, the PCR mixture had a final volume of 50 μL and contained 25 μL of Takara EmeraldAmp PCR Master Mix, 1 μL of each primer (20 pmol; Microsynth AG), 20 μL of PCR-grade water, and 3 μL of bacterial lysate. The PCR conditions were as follows: initial denaturation at 95 °C for 5 min, followed by 35 cycles of 95 °C for 1 min, 55 °C for 30 s, and 72 °C for 50 s, with a final extension at 72 °C for 10 min [20].

For *bla*SHV detection, the PCR mixture had a final volume of 50 μL and contained 25 μL of Takara EmeraldAmp PCR Master Mix, 1 μL of each primer (20 pmol; Microsynth AG), 21 μL of PCR-grade water, and 2 μL of bacterial lysate. The PCR conditions were as follows: initial denaturation at 94 °C for 5 min, followed by 30 cycles of 94 °C for 30 s, 68 °C for 1 min, and 72 °C for 1 min, with a final extension at 72 °C for 10 min [21].

All PCR amplification products were analyzed using the MCE-202 MultiNA microchip electrophoresis system for DNA/RNA analysis (Shimadzu Co., Kyoto, Japan), as shown in Appendix A, Figure A1, Figure A2, Figure A3 and Figure A4.

### 2.7. Detection of fimH, papC, hlylA, cnf1 and traT Virulence Genes

PCR amplification was performed to detect the *fim*H, *pap*C, *hly*A, *cnf*1, and *tra*T virulence genes in 159 FR *E. coli* O25 ST131 isolates, as previously described [22]. The primers used for gene detection are listed in Appendix B, Table A2.

Briefly, the PCR mixture had a final volume of 25 μL and contained 20 μL of Takara EmeraldAmp PCR Master Mix (Takara Bio, San Jose, CA, USA), 1 μL of each primer (20 pmol; Microsynth AG, Balgach, Switzerland), and 3 μL of bacterial lysate. The PCR conditions were as follows: initial denaturation at 95 °C for 5 min, followed by 25 cycles of 94 °C for 30 s, 63 °C for 30 s, and 68 °C for 3 min, with a final extension at 72 °C for 10 min.

All PCR amplification products were analyzed using the MCE-202 MultiNA microchip electrophoresis system for DNA/RNA analysis (Shimadzu Co., Kyoto, Japan), as shown in Appendix B, Figure A5, Figure A6, Figure A7, Figure A8 and Figure A9.

### 2.8. Serum and Serum Susceptibility Assay

Blood samples were obtained from three healthy volunteers and allowed to clot at room temperature for 30 min. Serum was separated by centrifugation at 1000× *g* for 15 min at 4 °C and subsequently pooled. An aliquot of the pooled serum was heat-inactivated at 56 °C for 30 min and used as a control.

Phenotypic serum susceptibility was assessed using the assay described by Schiller and Hatch [23]. Serum susceptibility was evaluated based on the reduction in colony-forming units (CFUs) following exposure to fresh human serum, reflecting complement-mediated bacterial killing.

Briefly, the bacterial suspension was standardized to approximately 10^4^ CFU/mL. An aliquot of 100 μL of the bacterial suspension was mixed with 100 μL of fresh, undiluted pooled human serum and incubated at 37 °C for 2 h. As a negative control, 100 μL of heat-inactivated serum was mixed with 100 μL of the bacterial suspension. Following incubation, samples were plated for CFU enumeration after overnight incubation at 37 °C. The survival percentage was calculated relative to the heat-inactivated serum control.

Serum resistance was defined as a reduction in CFU counts of <90%, indicating limited complement-mediated killing. Serum susceptibility was defined as a reduction in CFU counts of ≥90% relative to the heat-inactivated serum control, indicating effective serum bactericidal activity [23].

### 2.9. Statistical Analysis

Statistically significant differences in the research results were tested with the *χ*^2^-square test with the help of 2 × 2 contingency tables with the application of Yates correction. The value *p* < 0.05 was considered significant. 

In the descriptive statistics for metabolic potential results, absolute numbers and percentages were used to present categorical variables, while for numerical variables, median and range were used due to deviation from normal distribution. In the bivariate analysis of metabolic potential data, the *χ*^2^-square test or Fisher’s exact test (adjusted according to the frequency of expected frequencies in the contingency table) for categorical variables and the Mann–Whitney U test for numerical variables were used. Linear regression was also used for data analysis, with the dependent variable being the metabolic average while the independent variables were year of isolation and ESBL status (yes/no). The regression model showed a good fit with a Durbin–Watson test of 1.911 and an adjusted *R*^2^ of 7.1%. Statistical analysis was performed using the IBM SPSS Statistics program (v21.0; IBM, Armonk, NY, USA) and results with *p* < 0.05 were considered statistically significant.

## 3. Results

### 3.1. ESBL Phenotype and Detection of CTX-M, CTX-M-15, and TEM β-Lactamases Genes 

Among the uropathogenic *E. coli* isolates collected during the first and second study periods, 4354 and 7717 isolates, respectively, the proportion of ESBL-positive isolates ranged from 4.8% to 7.0%.

Among the 519 fluoroquinolone-resistant (FR) uropathogenic *E. coli* isolates collected during the two study periods, 48/151 (31.8%) isolates from the first study period and 122/368 (33.1%) from the second study period were ESBL-positive, with no statistically significant difference between the periods (*p* > 0.05). Among the O25 ST131 isolates (*n* = 159), ESBL-positivity rates were 66.7% in the first and 65.8% in the second study period. Similarly, among the 170 O25 isolates, ESBL positivity was detected in 30/48 (62.5%) and 75/122 (61.5%) of isolates, respectively, during the same periods (*p* > 0.05).

The frequencies of *bla*CTX-M, *bla*CTX-M-15, and *bla*TEM genes detected among the 105 ESBL-positive FR *E. coli* O25 ST131 isolates collected during both study periods are shown in Figure 1.

All investigated ESBL-positive O25 ST131 isolates carried the *bla*CTX-M gene. The *bla*CTX-M-15 gene was detected in 27 (90%) isolates from the first study period; among these, 12 (44%) also carried the IS26 insertion sequence upstream of *bla*CTX-M. In the second study period, *bla*CTX-M-15 was detected in 66 (88%) ESBL-positive O25 ST131 isolates, while IS26 was detected upstream of *bla*CTX-M in 12 (18%) isolates. No statistically significant difference was observed in the frequency of *bla*CTX-M-15 detection between the two study periods (*χ*^2^ = 0.08, *p* > 0.05).

The *bla*TEM gene was detected in four FR O25 ST131 ESBL-positive *E. coli* isolates from the first study period and in 13 isolates from the second study period. The simultaneous presence of *bla*TEM and *bla*CTX-M-15 was observed in two isolates from the first study period and in 12 isolates from the second study period. None of the ESBL-positive isolates carried the *bla*SHV gene during either study period.

### 3.2. Virulence Genes fimH, papC, hlylA, cnf1 and traT in O25 ST131 Isolates

The *fimH* gene was detected in all 159 isolates (100%), whereas *papC*, *hlyA*, *cnf1*, and *traT* were detected in 43 (27%), 35 (22%), 35 (22%), and 123 (77%) isolates, respectively.

During the first study period, *papC* and *hlyA* were detected in one isolate each, *cnf1* in two isolates, and *traT* in 33 isolates. During the second study period, *papC* was detected in 42 isolates, *hlyA* in 34, *cnf1* in 33, and *traT* in 90 isolates.

A statistically significant difference was observed between the two study periods in the detection of *papC*, *hlyA*, and *cnf1* (*χ*^2^ = 13.71, *p* < 0.01), with these virulence-associated genes detected more frequently during the second study period (Figure 2). In the first study period, only one of 45 isolates was *papC*-positive, compared with 42 of 114 isolates in the second study period. A similar pattern was observed for *hlyA* and *cnf1*. Only one *hlyA*-positive and two *cnf1*-positive isolates were detected among the isolates from the first study period.

A statistically significant difference was also observed in the distribution of *papC*, *hlyA*, and *cnf1* between ESBL-positive and ESBL-negative *E. coli* O25 ST131 isolates (*p* < 0.01) during both study periods. The simultaneous presence of these virulence genes was detected in 34 *E. coli* O25 ST131 isolates with an ESBL phenotype, including one isolate from the first study period and 33 from the second study period. In the second study period, one ESBL-negative *E. coli* O25 ST131 isolate also carried *fimH*, *papC*, *hlyA*, and *cnf1* simultaneously.

Overall, 35 *E. coli* O25 ST131 isolates carried *fimH*, *papC*, *hlyA*, and *cnf1* simultaneously, and *traT* was detected in 33 of these 35 isolates. The distribution of virulence-associated genes differed between the two study periods, with a higher frequency of *papC*, *hlyA*, and *cnf1* among isolates from the second study period and an association between these virulence profiles and the ESBL phenotype (*p* < 0.01).

### 3.3. Metabolic Activity of FR O25 ST131 Isolates During the Two Study Periods

Metabolic potential was calculated as the proportion of positive biochemical reactions among the 47 biochemical reactions assessed using the VITEK 2 GN card and was expressed both as the number of positive reactions and as the corresponding proportion (%).

During the first study period, the mean number of positive biochemical reactions was 17 (range, 16–19) among ESBL-negative *E. coli* O25 ST131 isolates. Among ESBL-positive isolates, the mean number of positive reactions was 18 (range, 14–21) out of 47 tested reactions.

During the second study period, the mean number of positive biochemical reactions was 19 (range, 14–21) among ESBL-negative *E. coli* O25 ST131 isolates and 19 (range, 14–23) among ESBL-positive isolates. The distribution of metabolic potential values is presented in Table 1.

The biochemical profiles of *E. coli* O25 ST131 isolates were compared between the two study periods based on the proportion of positive reactions for each individual biochemical substrate. Statistical significance was assessed using the chi-square test and Fisher’s exact test. The *p*-values for individual substrates comparing the two study periods are presented in Appendix C, Table A3.

Regression analysis demonstrated a statistically significant difference in metabolic potential between the two study periods (*p* < 0.01), whereas ESBL status (negative vs. positive) was not a statistically significant predictor of metabolic potential (*p* = 0.093; Table 2).

No statistically significant differences in metabolic potential according to ESBL status were observed within either study period or when comparing ESBL-positive and ESBL-negative isolates across the two study periods (*p* > 0.05).

### 3.4. Serum Susceptibility of Isolates During the Two Study Periods

A total of 89/159 (56.0%) *E. coli* O25 ST131 isolates demonstrated resistance to the bactericidal activity of serum. The proportion of serum-resistant (SR) isolates was 18/45 (40.0%) during the first study period and 71/114 (62.3%) during the second study period.

A statistically significant difference in the frequency of serum-resistant isolates was observed between the two study periods, with a higher proportion of SR isolates in the second period (*χ*^2^ = 6.50, *p* = 0.01).

No statistically significant association was observed between serum resistance and ESBL phenotype among *E. coli* O25 ST131 isolates (*p* > 0.05).

The presence of the *traT* gene, which has been associated with resistance to serum bactericidal activity, did not consistently correspond to the phenotypic serum susceptibility results. Overall, *traT* was detected in 123/159 (77.4%) isolates. The gene was detected in 33/45 (73.3%) isolates from the first study period and 90/114 (78.9%) isolates from the second study period, with no statistically significant difference between the periods (*p* > 0.05).

Among the 123 *traT*-positive isolates, 49 (39.8%) were serum-susceptible and 74 (60.2%) were serum-resistant. In the first study period, 20 serum-susceptible and 13 serum-resistant isolates carried *traT*, whereas in the second study period, 29 serum-susceptible and 61 serum-resistant isolates carried *traT*. The presence of *traT* was significantly associated with the serum susceptibility phenotype (*χ*^2^ = 3.87, *p* = 0.049).

The distribution of serum-susceptible and serum-resistant *E. coli* O25 ST131 isolates is presented in Appendix C, Table A4.

### 3.5. Patients’ Demographic Characteristics

FR *E. coli* isolates were recovered from 124 female and 27 male patients (male-to-female ratio, 1:4.6) aged 11–95 years during the first study period and from 288 female and 80 male patients (male-to-female ratio, 1:3.6) aged 9–98 years during the second study period.

The O25 ST131 clone was detected in 39 female and six male patients aged 14–95 years during the first study period and in 83 female and 31 male patients aged 9–93 years during the second study period. The mean age was 69 years among ESBL-positive patients in the first study period and 72 years in the second study period. No statistically significant difference was observed between the two study periods (*χ*^2^ = 0.07, *p* > 0.05).

A limitation of this retrospective study was the lack of clinical information beyond patient demographic characteristics.

Preliminary results were presented in a poster session at the 35th European Congress of Clinical Microbiology and Infectious Diseases (ECCMID), held in Vienna, Austria, from 27 to 30 April 2025 (Anušić M, Vujnović T, Vlahek L, Tičić V, Kolčić I, Marijan T, Bedenić B, Vraneš J. Virulence and fitness of CTX-M-producing *Escherichia coli* O25 ST131 in community-acquired urinary tract infections in Croatia).

## 4. Discussion

The pandemic clone *E. coli* O25 ST131 is divided into three major genetic clades (A, B, and C). Most contemporary *E. coli* O25 ST131 isolates belong to clade C, which is characterized by the *fimH30* allele encoding the type 1 fimbrial adhesin and is further divided into the H30-R (fluoroquinolone-resistant) and H30-Rx (fluoroquinolone-resistant and ESBL-producing) subclades, corresponding to C1 and C2, respectively. Type 1 fimbriae are recognized as an important virulence factor in uropathogenic *E. coli* (UPEC).

In our study, all *E. coli* O25 ST131 isolates carried the *fimH* gene, whereas *papC*, *hlyA*, and *cnf1* were detected significantly more frequently during the second study period. Virulence-associated genes present in the *E. coli* O25 ST131 clonal lineage have been used to define several virotypes (A–G). Virotype A is characterized by the presence of *afa/draBC* and an *afa* operon gene, with the *sat* gene being variably present. Virotype B is characterized by the presence of *iroN*, with *sat* being variably present, whereas virotype C is characterized by the presence of *sat*. Virotype D is characterized by variable presence of *afa/draBC*, *afa* operon, *iroN*, *sat*, *cnf1*, *hlyA*, *papGIII*, *cdtB*, and *neuC-K1*, together with the presence of *ibeA*. Virotype E is characterized by the presence of *sat*, *papGII*, *cnf1*, and *hlyA*, whereas virotypes F and G are characterized by the presence of *iroN*, *papGII*, and *cnf1*, and *sat* and *papGII*, respectively [24,25].

Calhau et al. demonstrated the presence of pathogenicity islands PAI I CFTO73, PAI II CFTO73, and PAI IV 536 in *E. coli* ST131 isolates, containing virulence determinants associated with P fimbriae, siderophores, and α-hemolysin [26]. In our study, the *fimH*/*papC*/*hlyA*/*cnf1*/*traT* virulence-associated gene profile predominated among FR *E. coli* O25 ST131 ESBL-positive isolates during the second study period. The simultaneous presence of *papC*, *hlyA*, and *cnf1* was detected in 33 of 75 ESBL-positive isolates during the second study period, compared with one of 39 ESBL-negative isolates. Further studies are required to determine whether these isolates correspond to virotype E and to assess the presence of the relevant pathogenicity islands, as well as their potential contribution to UTI development in outpatients without known predisposing factors.

ESBL production has frequently been associated with reduced virulence compared with ESBL-negative *E. coli* isolates. However, Banerjee et al. reported that the ESBL-positive *E. coli* ST131 clonal lineage carried a greater number of characterized virulence genes than non-ST131 *E. coli* isolates [27]. Similarly, our findings are consistent with those reported by Hojabri et al., who found that ESBL-positive *E. coli* ST131 H30Rx isolates were more frequently associated with *papA*, *papC*, *papEF*, *hlyA*, *cnf1*, *k5*, and *kpsMT II*, and with the findings of Gibreel et al., who reported a higher number of virulence genes among CTX-M-15-producing UPEC ST131 isolates than among non-ST131 isolates [28,29].

In our study, ESBL-positive *E. coli* O25 ST131 isolates carried a higher number of the selected virulence-associated genes than ESBL-negative isolates. These findings suggest an association between ESBL production and the presence of the selected virulence-associated genes in *E. coli* O25 ST131. However, the presence of virulence-associated genes does not necessarily demonstrate increased phenotypic virulence, and functional and clinical studies are required to establish whether these genetic differences translate into differences in pathogenic potential.

The O25 ST131 *E. coli* clonal lineage requires metabolic adaptation to transition from the gastrointestinal tract to the urinary tract and to respond to changes in pH and nutrient availability. The metabolic findings from our study can be compared with those of two studies conducted in England. Gibreel et al. reported significantly higher metabolic potential among O25 ST131 isolates than among other sequence types, including ST10, ST14, ST69, ST73, ST88, ST95, ST127, and ST405. In that study, O25 ST131 was significantly associated with six positive biochemical reactions—AGAL (α-galactosidase), BGUR (β-glucuronidase), ODC (ornithine decarboxylase), ProA (L-proline arylamidase), SAC (sucrose/sucrose), and ILATk (L-lactate alkalinization)—and with two negative biochemical reactions, ILATa (L-lactate assimilation) and 5KG (5-keto-D-gluconate), using the VITEK 2 ID GNB card [15].

In the same study, comparisons of susceptible and multidrug-resistant *E. coli* O25 ST131 isolates showed that CTX-M-15-producing isolates had significantly higher metabolic potential. The biochemical characteristics associated with CTX-M-15-producing *E. coli* O25 ST131 included positive reactions for tyrosine arylamidase, L-lactate alkalinization, phosphatase, and succinate alkalinization. Cluster analysis of biochemical profiles identified a large cluster containing multidrug-resistant *E. coli* O25 ST131 isolates [15].

In contrast, Alqasim et al., using four API kits and the Biolog automated system, did not demonstrate differences in metabolic potential between *E. coli* ST131 and other UPEC sequence types, including ST69, ST73, ST95, and ST10. In that study, ST131 isolates showed low utilization of five substrates—5KG (8%), D-arabinose (20%), esculin (24%), cellobiose (12%), and dulcitol (36%)—compared with other sequence types. Genome sequence analysis also showed little difference in the presence or absence of metabolic operons between ST131 and other sequence types. The authors therefore concluded that *E. coli* ST131 was not associated with a distinct biochemical profile and that observed differences were more likely related to individual ST131 strains than to the clonal lineage as a whole [30].

In our study, the metabolic potential of UPEC *E. coli* O25 ST131 isolates was evaluated during two study periods without comparison with other fluoroquinolone-resistant UPEC isolates. A total of 112 biochemical profiles were observed; therefore, a characteristic biochemical profile specific to *E. coli* O25 ST131 could not be established. Nevertheless, several biochemical reactions were comparable with those reported in previous studies. The negative reactions ILATa (L-lactate assimilation) and 5KG (5-keto-D-gluconate) reported by Gibreel et al. were also predominantly negative in our isolates [15]. In our study, ILATa was positive in 1.75% (2 isolates) during the first study period and 7.89% (9 isolates) during the second period, whereas 5KG was positive in only one isolate (0.87%) during the second period. D-cellobiosis was negative during both study periods, consistent with the findings of Alqasim et al. [30].

Of the six positive biochemical reactions associated with O25 ST131 by Gibreel et al.—AGAL, BGUR, ILATk, ODC, ProA, and SAC—all were also positive among the *E. coli* O25 ST131 isolates investigated in our study. Metabolic activity was significantly higher among isolates collected during the second study period than among those collected during the first period, whereas ESBL status did not significantly affect metabolic potential. Further studies are required to determine whether the observed temporal differences in metabolic activity are associated with bacterial fitness, including the ability to colonize the gastrointestinal tract and cause urinary tract infections.

Resistance to the bactericidal activity of serum is also considered a virulence-associated trait and may be particularly relevant to bacteria capable of invasive infection and hematogenous dissemination. The bactericidal activity of human and animal serum is mediated primarily by the complement system. Phan et al. investigated genes associated with serum resistance in EC958, a representative UPEC ST131 isolate, and identified strong associations with genes involved in the synthesis of the murein lipoprotein Lpp and A-lipids, as well as genes regulating O-antigen chain length [31].

In our study, the presence of the *traT* gene and the phenotypic susceptibility of FR *E. coli* O25 ST131 isolates to the bactericidal activity of human serum were assessed. The *traT* gene was detected in 83.1% (74/89) of serum-resistant isolates and in 70.0% (49/70) of serum-susceptible isolates (*p* < 0.05). In contrast, Kanukollu et al. reported no statistically significant difference in the prevalence of *traT* between serum-resistant and serum-susceptible *E. coli* isolates [32].

The precise mechanism by which TraT contributes to serum resistance remains incompletely characterized. It has been proposed that the TraT outer membrane lipoprotein may interfere with complement-mediated membrane damage [33]. Although we observed an association between the presence of *traT* and serum resistance, complete concordance was not observed, likely reflecting the contribution of additional mechanisms to resistance against the bactericidal activity of serum [34,35,36].

In our study, comparison of the phenotypic susceptibility of FR *E. coli* O25 ST131 isolates to serum bactericidal activity between the two study periods demonstrated a significantly higher frequency of serum-resistant isolates during the second period. The simultaneous presence of *fimH*, *papC*, *hlyA*, *cnf1*, and *traT* in serum-resistant isolates, particularly among ESBL-positive isolates during the second study period, suggests the occurrence of isolates carrying multiple virulence-associated traits. Further clinical studies are required to determine whether this combination of characteristics is associated with complicated urinary tract infections, bacteremia, or urosepsis.

Recent studies have reported that *E. coli* ST131 isolates carrying *bla*CTX-M genes, including *bla*CTX-M-15, may exhibit enhanced *in vitro* fitness and prolonged intestinal persistence, challenging the traditional assumption that antimicrobial resistance necessarily imposes a substantial fitness cost [37,38,39,40,41]. Type 1 fimbriae and other virulence-associated factors may contribute to efficient intestinal colonization. The evolution of *E. coli* O25 ST131 has therefore been characterized by the acquisition and maintenance of antimicrobial resistance and virulence-associated determinants, including fluoroquinolone resistance and ESBL genes [42,43,44]. These characteristics may contribute to the successful persistence and dissemination of this clonal lineage and its involvement in urinary tract and bloodstream infections.

The present study has several limitations. The analysis was restricted to two selected time periods, and the study population consisted of isolates recovered from outpatients with acute urinary tract infections. In addition, the analysis of virulence was based on the detection of selected genes rather than on functional assays of virulence. Therefore, the observed differences in gene prevalence should not be interpreted as direct evidence of differences in pathogenicity. Prospective studies incorporating broader genomic characterization, functional assays, and clinical outcome data are warranted to further investigate temporal changes in this high-risk clonal lineage.

## Figures and Tables

**Figure 1:**
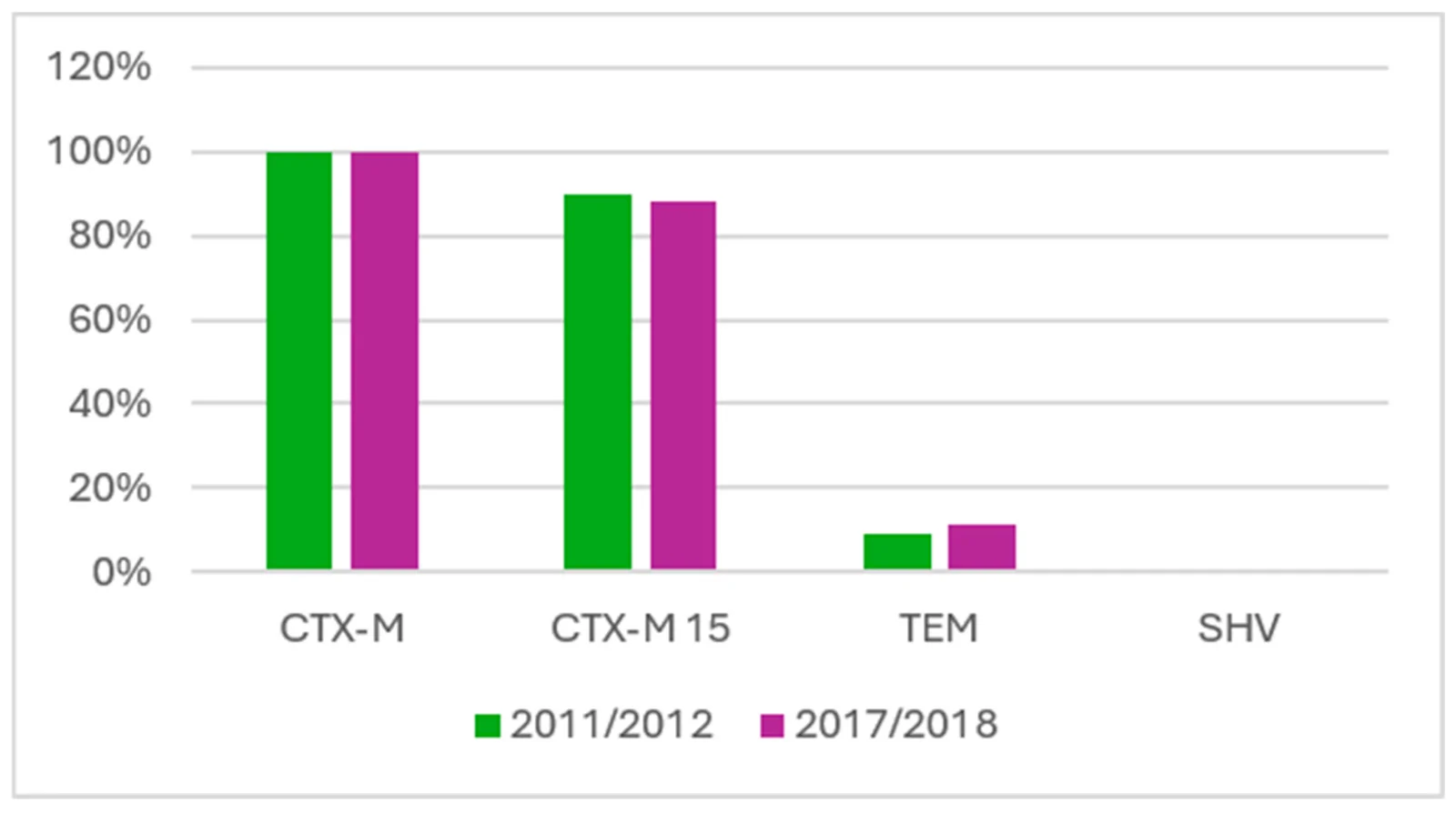
Detection of CTX-M, CTX-M-15 and TEM β-lactamases in 105 ESBL-positive FR O25 ST131 *E. coli* isolates.

**Figure 2:**
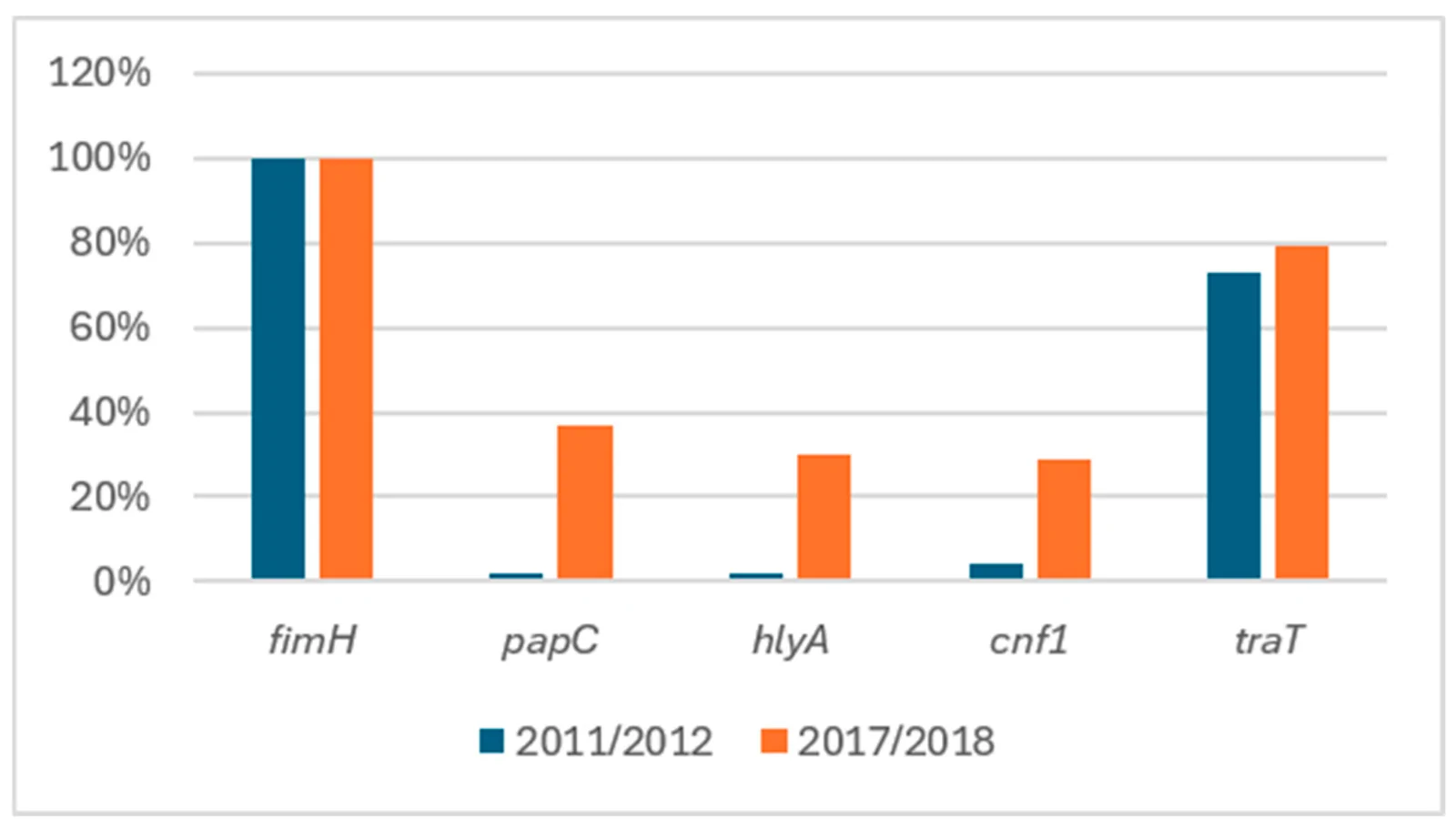
Detection of virulence genes in 159 FR O25 ST131 isolates during the two study periods.

**Table 1:** Metabolic potential of FR O25 ST131 *E. coli* isolates.

Metabolic Potential	2011/2012 *n* (%) Total 45 Isolates	2017/2018 *n* (%) Total 114 Isolates
0.48 (23/47)	0	1 (0.9)
0.46 (22/47)	0	2 (1.8)
0.44 (21/47)	3 (6.7)	8 (7)
0.42 (20/47)	1 (2.2)	28 (24.6)
0.40 (19/47)	12 (26.7)	26 (22.8)
0.38 (18/47)	8 (17.8)	22 (19.3)
0.36 (17/47)	10 (22.2)	19 (16.6)
0.34 (16/47)	8 (17.8)	3 (2.6)
0.32 (15/47)	1 (2.2)	3 (2.6)
0.30 (14/47)	2 (4.4)	2 (1.8)

**Table 2:** Linear regression analysis results, with the metabolic average as the dependent variable.

	Beta	95% CI	*p*-Value
Study year (2011 was the control group)	0.257	0.008–0.032	<0.001
ESBL status (negative was the control group)	0.129	−0.002–0.021	0.093

**Figure A1:**
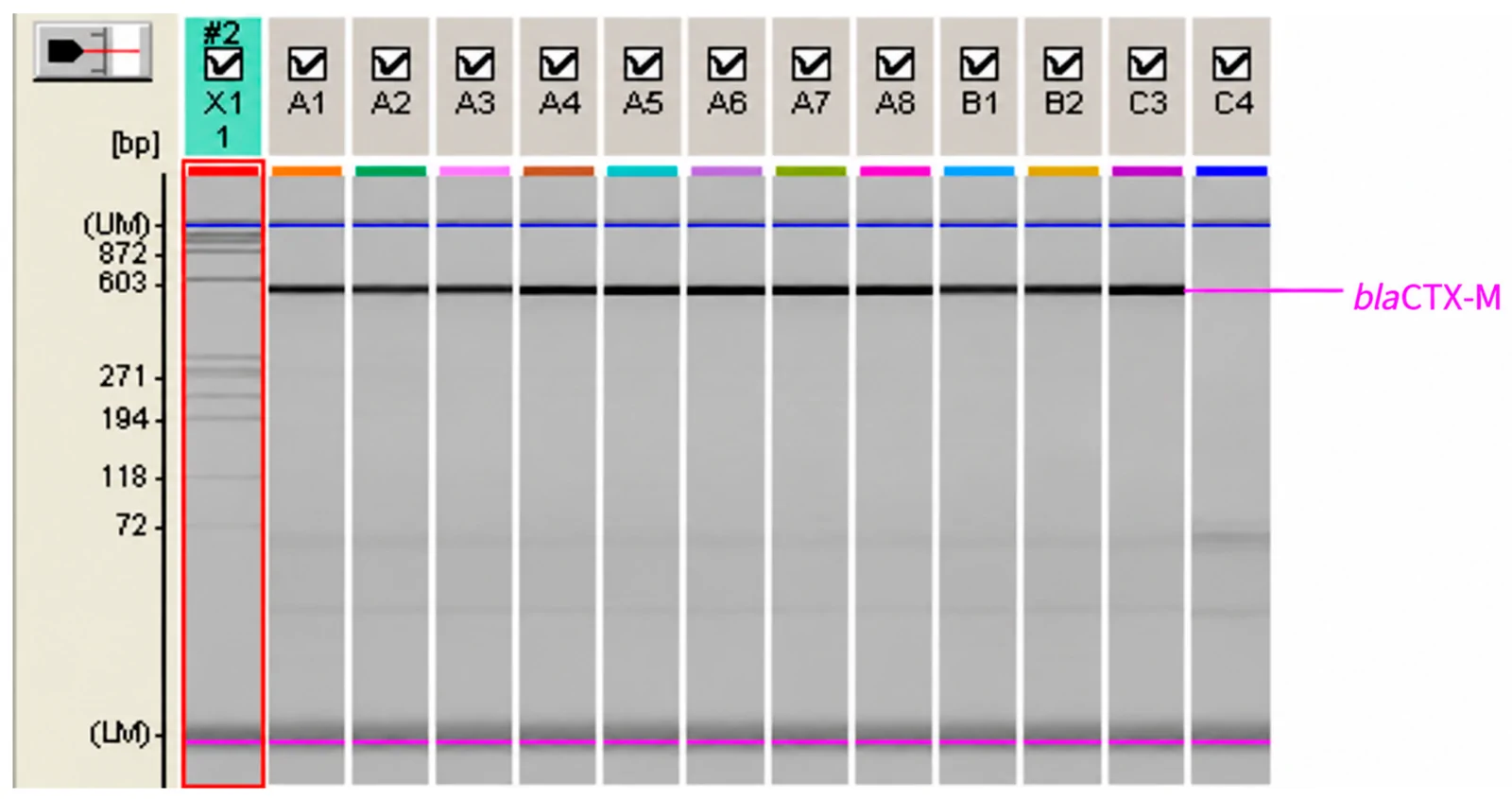
Microchip electrophoresis for the CTX-M gene (542 bp). DNA range bp—X1; positive samples—A1, A2, A3, A4, A5, A6, A7, A8, B1, B2; positive control *K. pneumoniae* CTX-M—C3; negative control *E. coli* ATCC 25922—C4.

**Figure A2:**
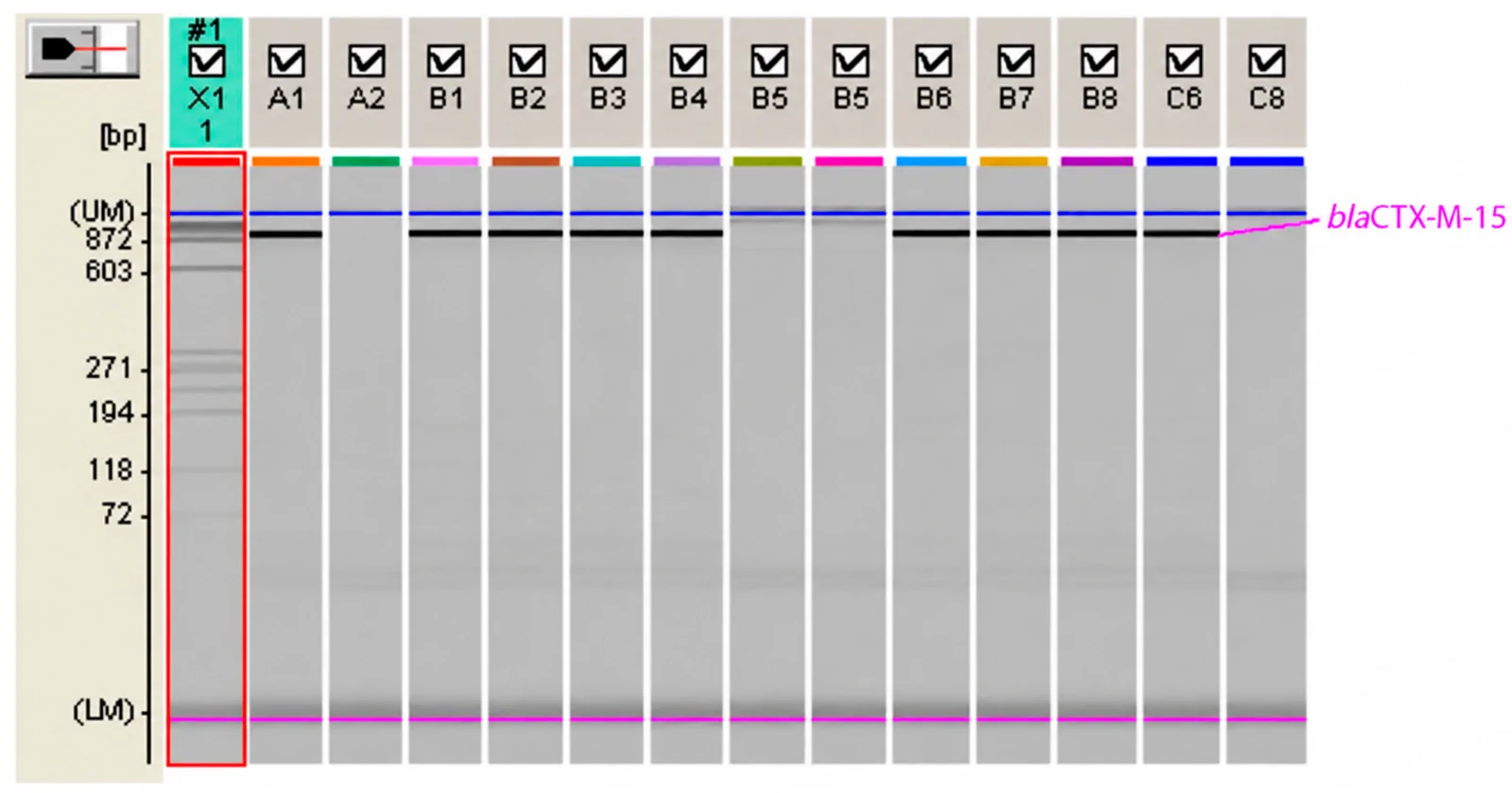
Microchip electrophoresis for the CTX-M-15 gene (1020 bp). DNA range bp—X1; positive samples—B1, B2, B3, B4, B7, B8, C6; negative samples—A2, B5, B6; positive control *E. coli* CTX-M-15—A1; negative control—*E. coli* ATCC 25922—C8.

**Figure A3:**
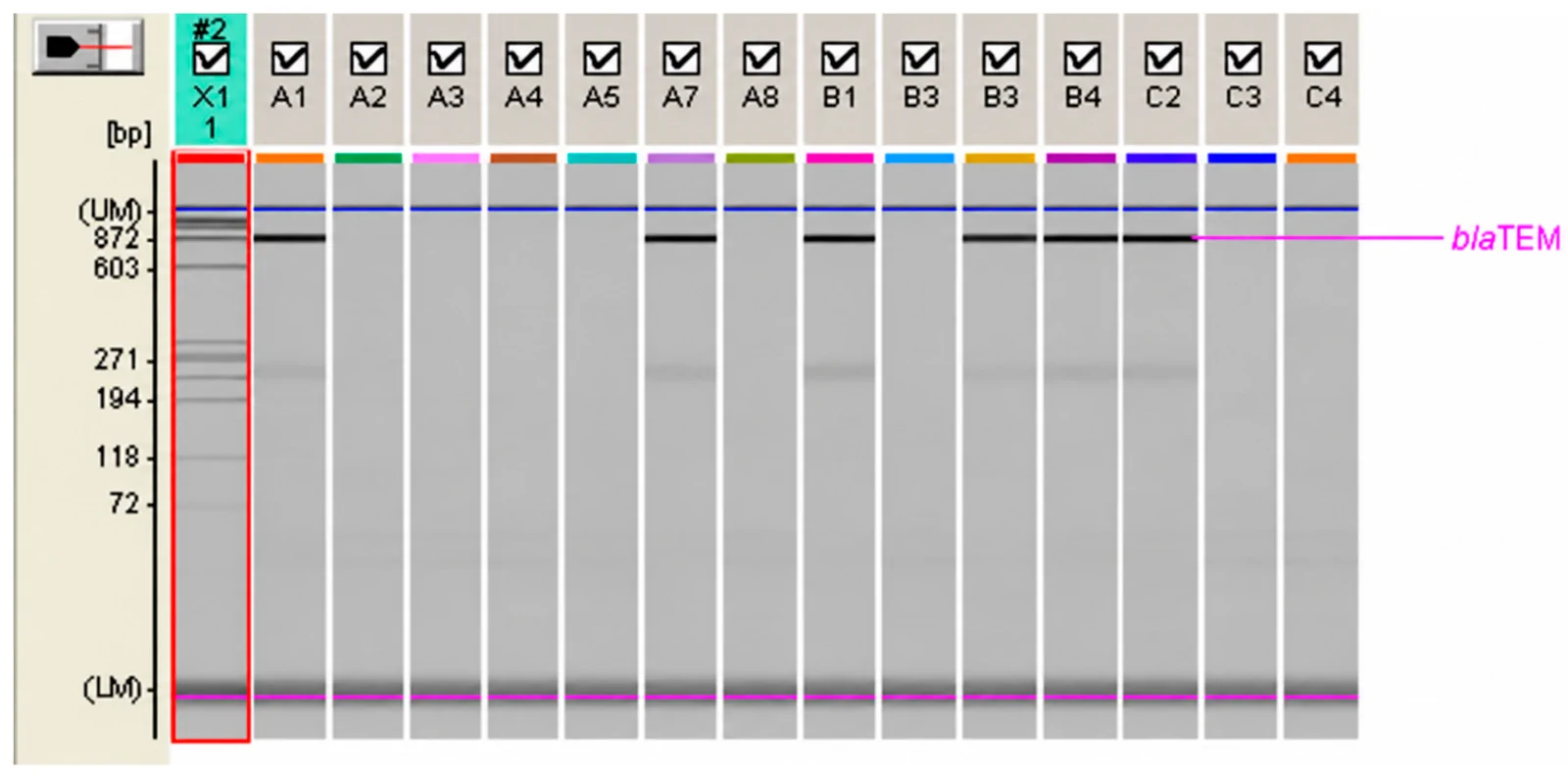
Microchip electrophoresis for the TEM gene (868 bp). DNA range bp—X1; positive samples—A1, A7, B1, B4; negative samples—A2, A3, A4, A5, A8, B3, C4; positive control TEM2 producing *E. coli*—C2, negative control *E. coli* ATCC 25922—C3.

**Figure A4:**
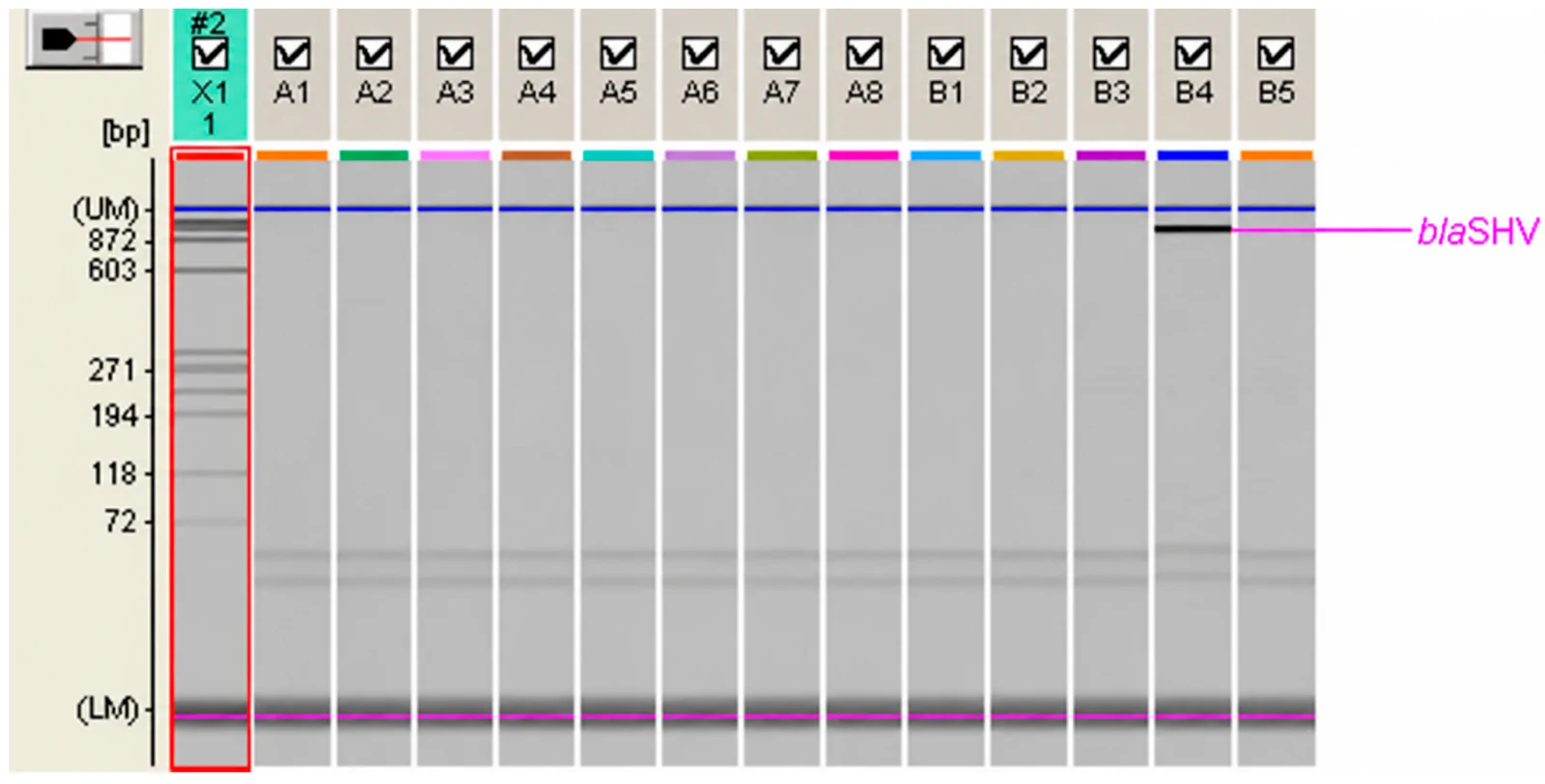
Microchip electrophoresis for the SHV gene (1127 bp). DNA range bp—X1; negative samples—A1, A2, A3, A4, A5, A6, A7, A8, B1, B; positive control SHV2a producing K. pneumoniae—B4; negative control E. coli ATCC 25922—B3, B5.

**Figure A5:**
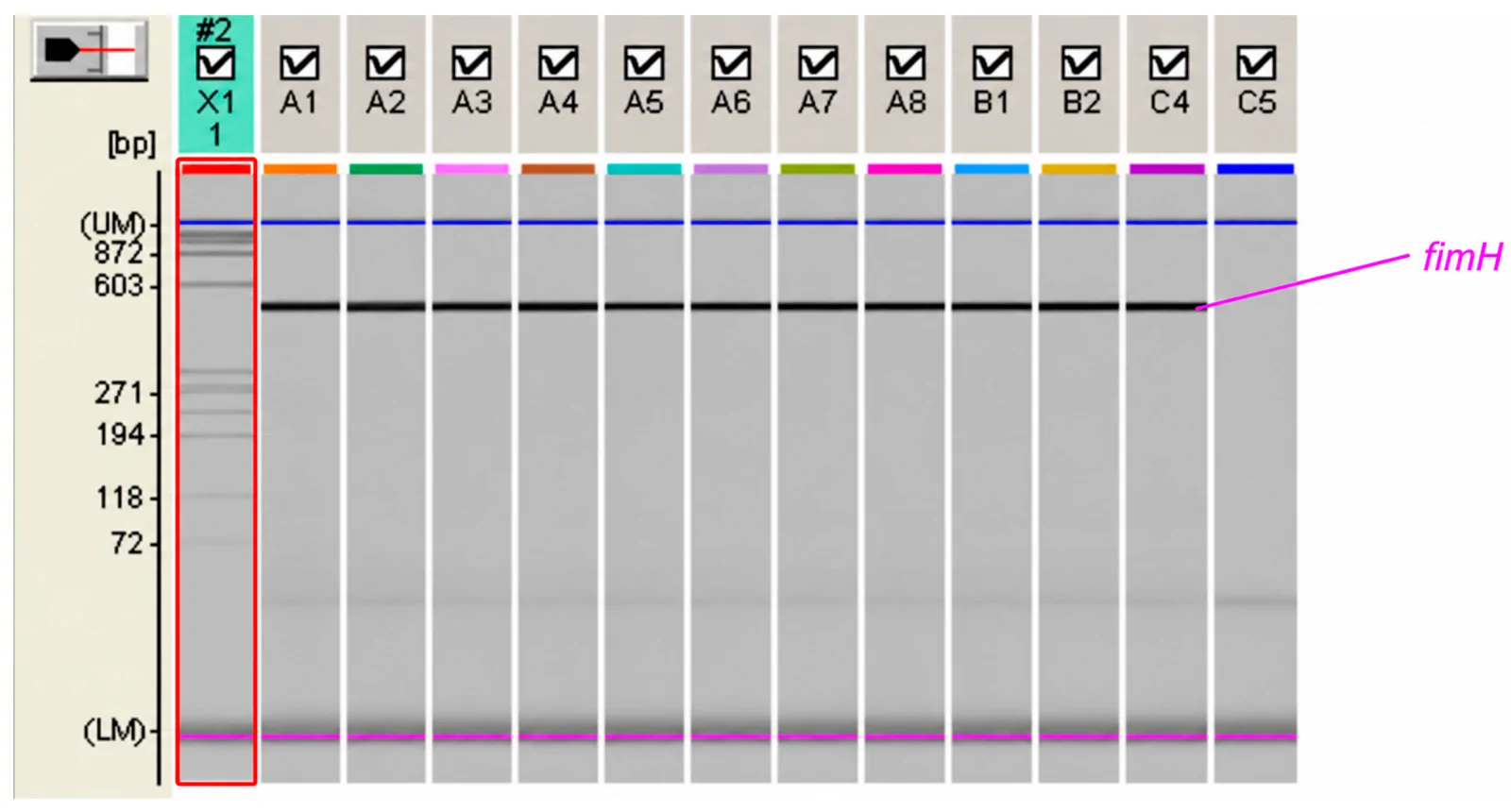
Microchip electrophoresis for the virulence gene *fimH* (508 bp). DNA range bp—X1; positive samples—A1, A2, A3, A4, A5, A6, A7, A8, B, B2; positive control—C4; negative control-C5.

**Figure A6:**
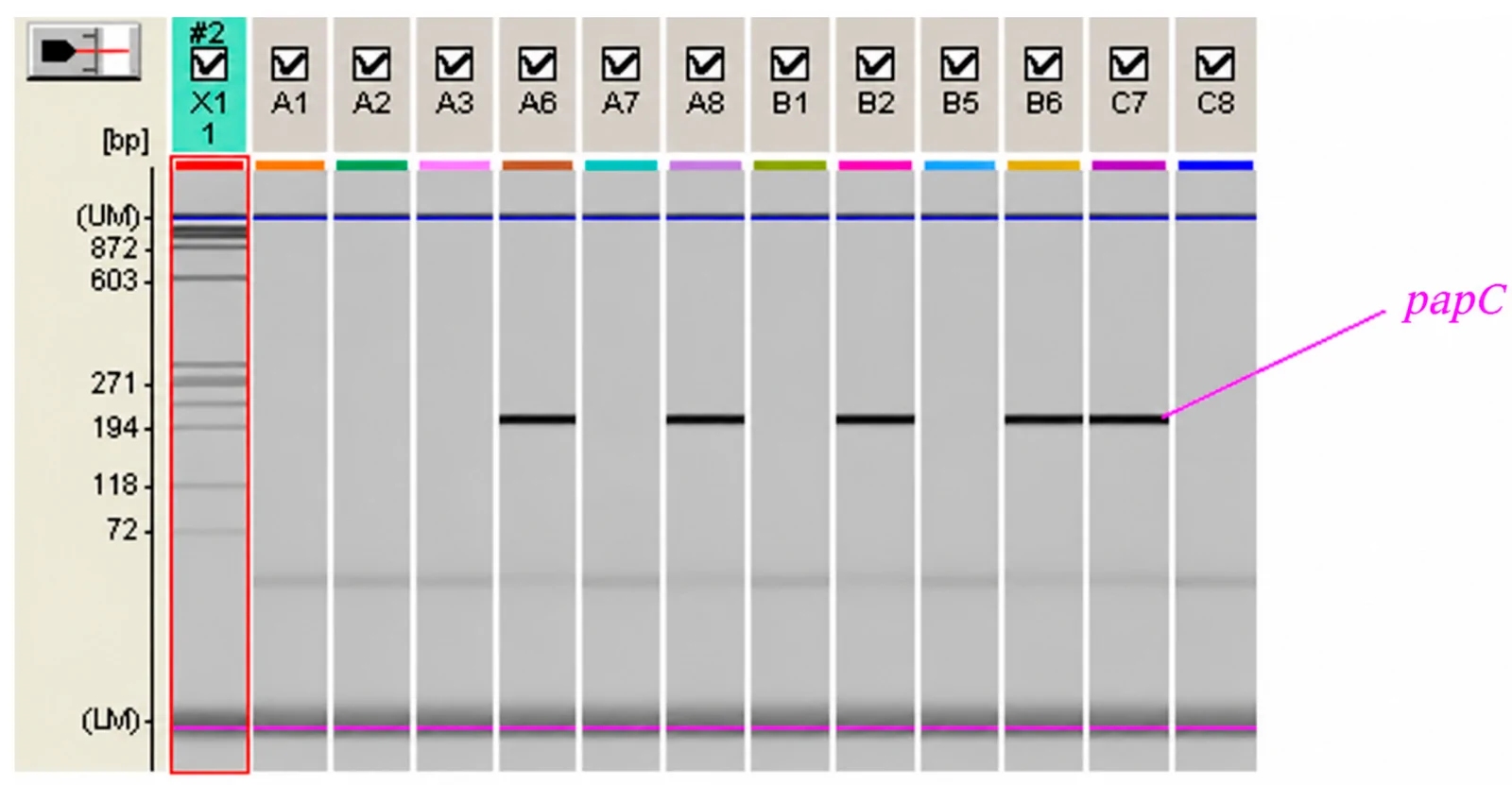
Microchip electrophoresis for the virulence gene papC (205 bp). DNA range bp—X1; positive samples—A6, A8, B2, B6; negative samples—A1, A2, A3, A7, B1, B5; positive control—C7; negative control—C8.

**Figure A7:**
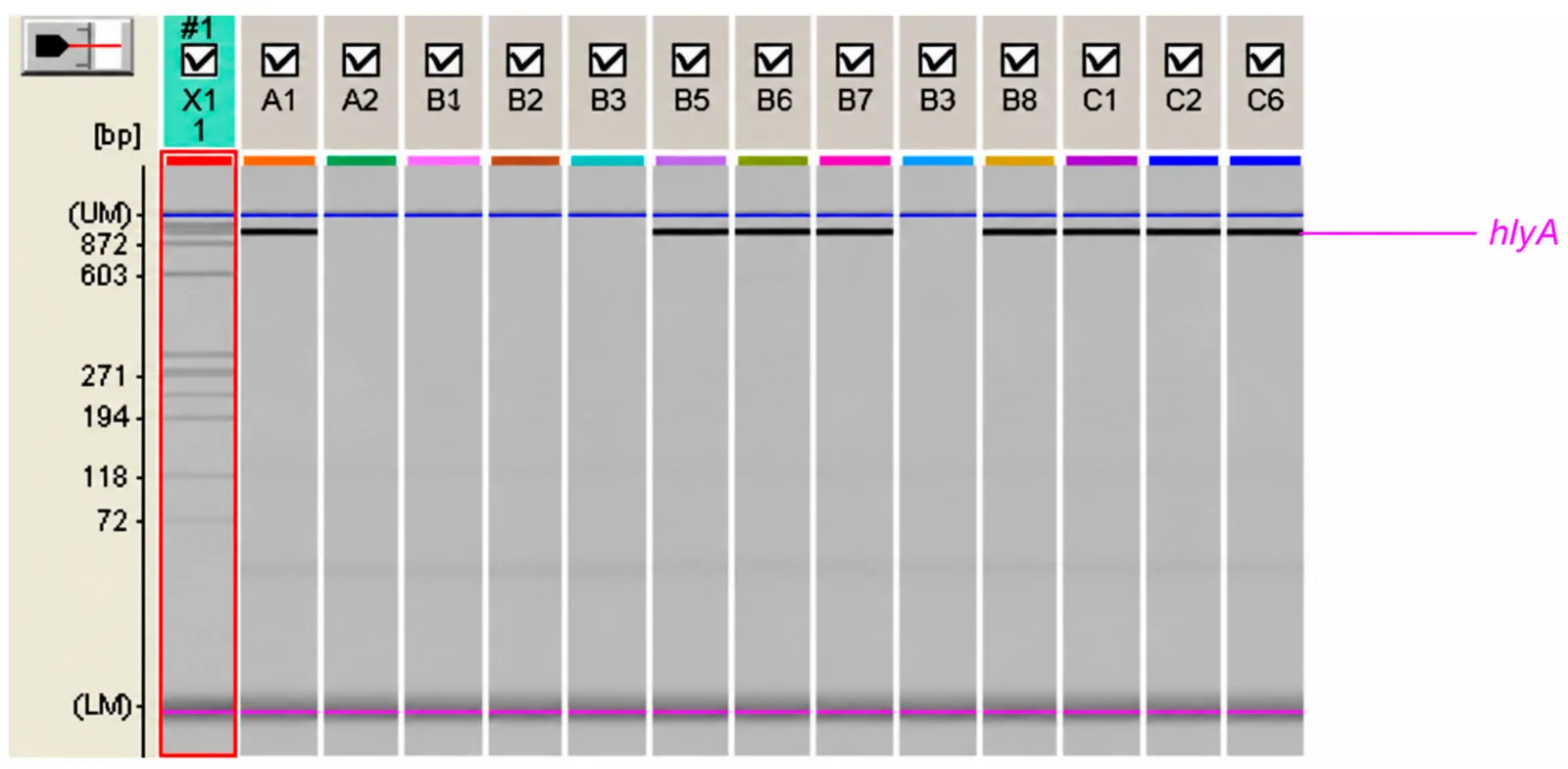
Microchip electrophoresis for the virulence gene *hly*A (1257 bp). DNA range bp—X1; positive samples—B5, B6, B7, C1, C2, C6; negative samples—B1, B2, B3, B8; positive control—A1, negative control—A2.

**Figure A8:**
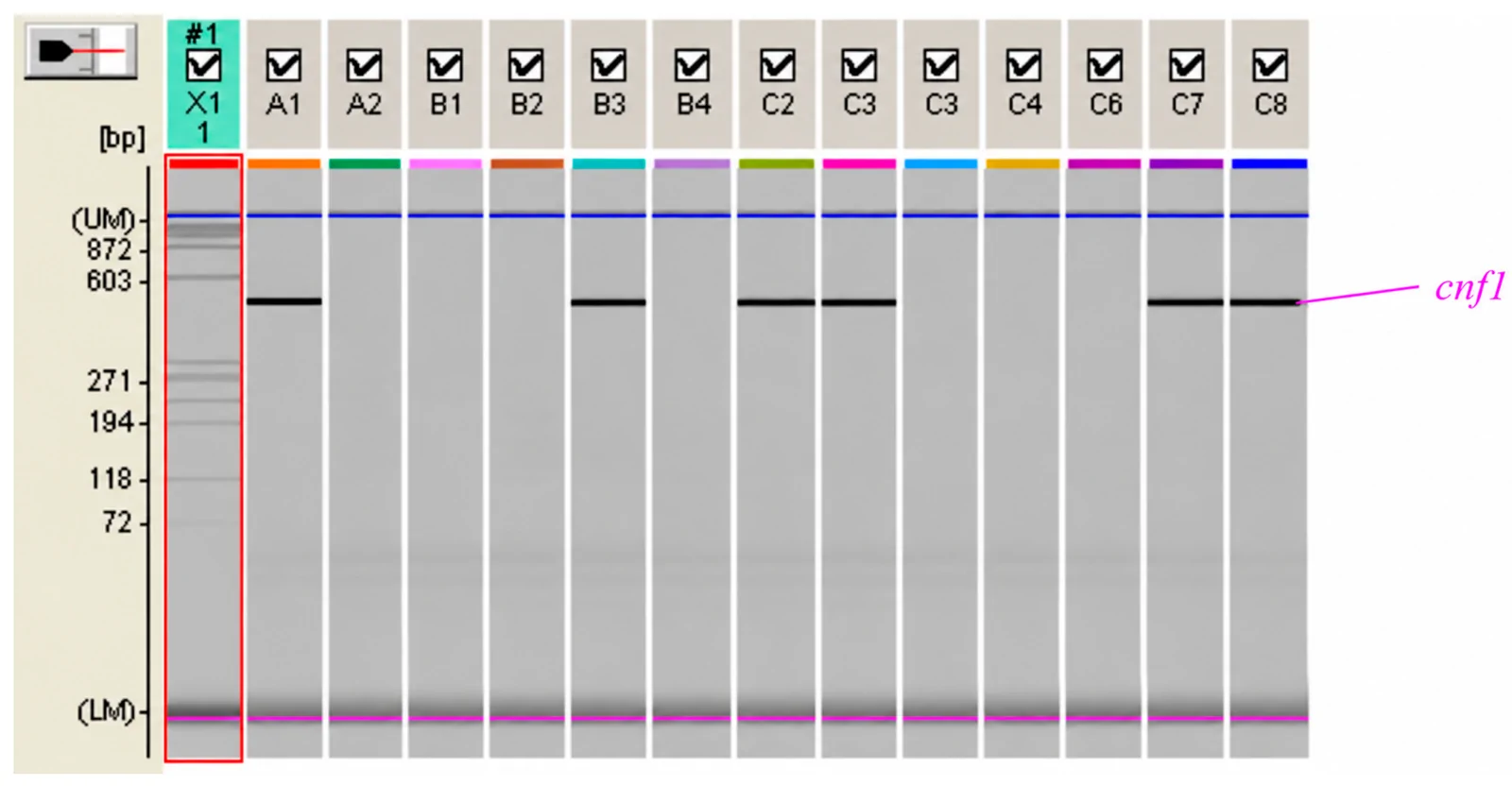
Microchip electrophoresis for the virulence gene *cnf*1 (498 bp). DNA range bp—X1; positive samples—B3, C2, C3, C7, C8; negative samples—B1, B2, B4, C4, C6; positive control—A1; negative control—A2.

**Figure A9:**
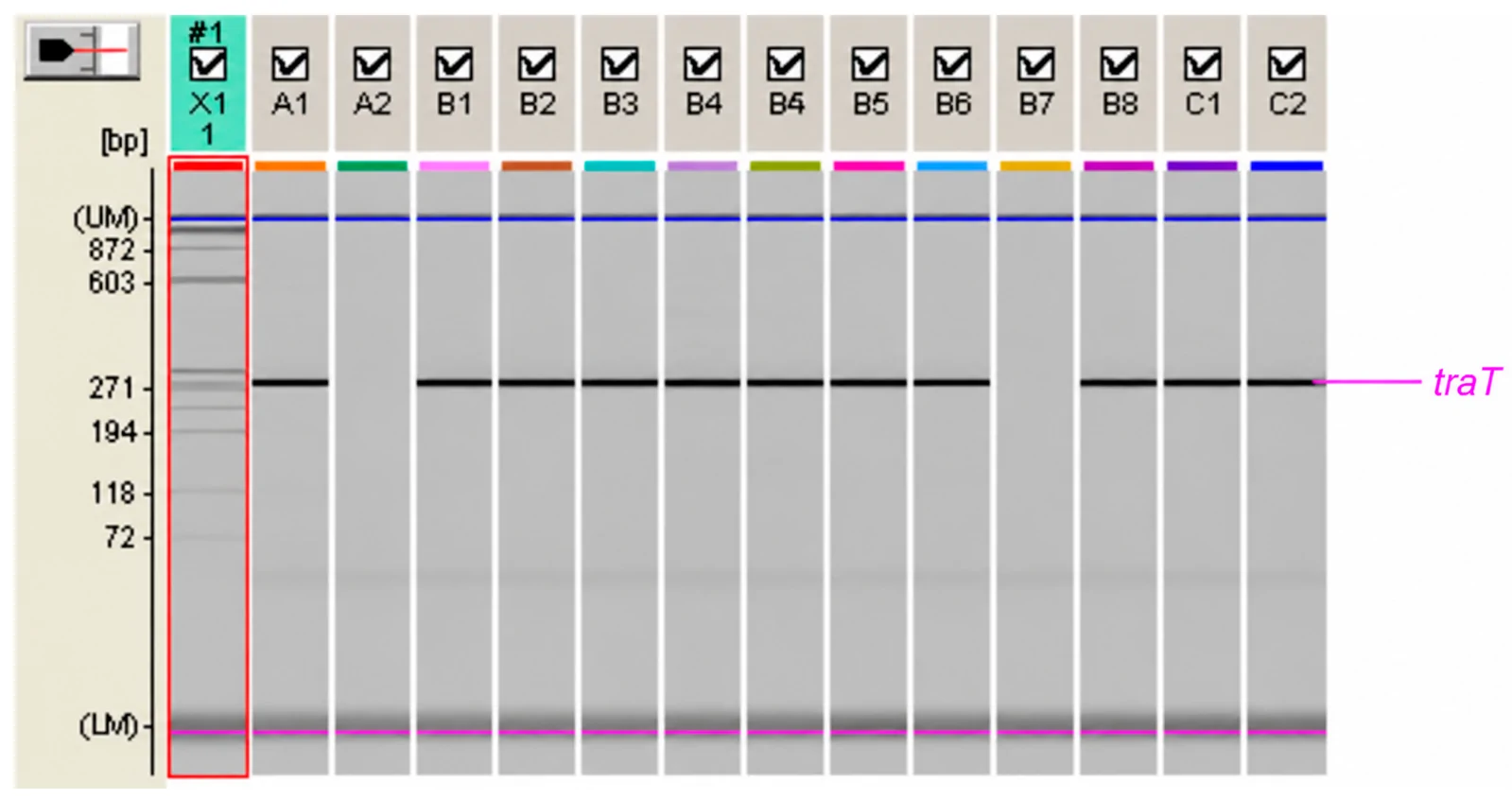
Microchip electrophoresis for the virulence gene *tra*T (290 bp). DNA range bp—X1; positive samples—B1, B2, B3, B4, B5, B6, B7, C1, C2; negative samples—B8; positive control—A1; negative control—A2.

## Data Availability

The original contributions presented in this study are included in the article. Further inquiries can be directed to the corresponding author.

## Abbreviations

The following abbreviations are used in this manuscript:

AMR	Antimicrobial resistance
MDR	Multidrug resistant
ExPEC	Extraintestinal pathogenic *Escherichia coli*
UTI	Urinary tract infection
UPEC	Uropathogenic *Escherichia coli*
CA-UTI	Community-acquired urinary tract infection
ESBL	Extended-spectrum beta-lactamases
EUCAST	European Committee on Antimicrobial Susceptibility Testing
PCR	Polymerase chain reaction
SS	Serum-susceptible
SR	Serum-resistant
CFU	Colony-forming units
TEM	Temoneira beta-lactamase
SHV	Sulfhydryl reagent variable beta-lactamase
CTX-M	CefoTaXimase first isolated in Munich
FR	Fluoroquinolone resistant
MLST	MultiLocus Sequence Typing
ST	Sequence type
APPA	Ala-Phe-Pro-arylamidase
H2S	H2S production
BGLU	Beta-glucosidase
PROA	L-Proline Arylamidase
SAC	Saccharose/sucrose
ILATK	L-lactate alkalinization
GLYA	Glycine arylamidase
O129R	O/129 resistance (comp.vibrio.)
ADO	Adonitol
BNAG	Beta-N-acetyl-glucosaminidase
DMAL	D-maltose
LIP	Lipase
IARL	L-arbitol
DGLU	D-glucose
DMNE	D-mannose
TYRA	Tyrosine arylamidase
CIT	Citrate
NAGA	Beta-N-acetyl-galactosaminidase
IHISA	L-histidine assimilation
ELLM	Ellman
DCEL	D-cellobiose
GGT	Gamma-glutamyl-transferase
BXYL	Beta-xylosidase
URE	Urease
DTAG	D-tagatose
AGLU	Alpha-glucosidase
ODC	Ornithine decarboxylase
GGAA	Glu-Gly-Arg-arylamidase
PYRA	L-pyrrolydonyl-arylamidase
AGLTP	Glutamyl arylamidase pNA
DMAN	D-mannitol
PLE	Palatinose
DTRE	D-trehalose
SUCT	Succinate alkalinization
LDC	Lysine decarboxylase
IMLTA	L-malate assimilation
MNT	Malonate
AGAL	Alpha-galactosidase
CMT	Coumarate
ILATA	L-lactate assimilation
BGAL	Beta-galactosidase
OFF	Fermentation/glucose
BAIAP	Beta-alanine-arylamidase pNA
DSOR	D-sorbitol
5KG	5-keto-D-gluconate
PHOS	Phosphatase
BGUR	Beta-glucuronidase

## Appendix A

**Table A1:** Selected primers used in the study.

Genes	Sequence of the Primer	Amplification Product (bp)	References
*bla*CTX-M	F: 5′-ATGTGCAGCACCAGTAAAGT-3′	545	[17]
*bla*CTX-M	R: 5′-ACCGCGATATCGTTGGTGG-3′		
*bla*CTX-M-15	F: 5′-CACACGTGGAATTTAGGGACT-3′	1020	[19]
*bla*CTX-M-15	R: 5′-GCCGTCTAAGGCGATAAACA-3′		
*bla*TEM	F: 5′-ATGAGTATTCAACATTTCCG-3′	858	[20]
*bla*TEM	R: 5′-CCAATGCTTAATCAGTGAGG-3′		
*bla*SHV	F: 5′-GCCCGGGTTATTCTTATTTGTCGC-3′	1017	[21]
*bla*SHV	R: 5′-CTTTCCGATGCCGCCGCCAGTCA-3′		

## Appendix B

**Table A2:** Primers used for the detection of *fimH*, *papC*, *hlyA*, *cnf1* and *traT* genes.

Gene	Sequence of Primer	Amplification Product (bp)	Reference
*fimH*	F: 5′-TGCAGAACGGATAAG CCG TGG-3′	508	[22]
*fimH*	R: 5′-GCAGTCACCTGCCCTCCGGTA-3′		
*papC*	F: 5′-GTGGCAGTATGAGTAATGACCGTTA-3′	205	[22]
*papC*	R: 5′-ATATCCTTTCTGCAGGGATGCAATA-3′		
*hlyA*	F: 5′-AACAAGGATAAGCACTGTTCTGGCT-3′	1257	[22]
*hlyA*	R: 5′-ACCATATAAGCGGTCATTCCCGTCA-3′		
*cnf1*	F: 5′-AAGATGGAGTTTCCTATGCAGGAG-3′	498	[22]
*cnf1*	R: 5′-CATTCAGAGTCCTGCCCTCATTATT-3′		
*traT*	F: 5′-GGTGTGGTGCGATGAGCACAG-3′	290	[22]
traT	R: 5′-CACGGTTCAGCCATCCCTGAG-3′		

## Appendix C

**Table A3:** Comparison of biochemical reactions of FR *E. coli* O25 ST131 isolates in two study periods.

Biochemical Substrate	Biochemical Reactions *n* (%) 2011/2012	Biochemical Reactions *n* (%) 2017/2018	*p*-Value
APPA	0	0	-
H2S	0	0	-
BGLU	0	0	-
PROA	8 (17.77)	37 (32.45)	*p* = 0.064
SAC	45 (100)	113 (99.12)	*p* = 1.000
ILATK	23 (51.11)	90 (78.94)	***p*** **= 0.000**
GLYA	2 (4.44)	8 (7.01)	*p* = 0.726
O129R	26 (57.77)	76 (66.66)	*p* = 0.292
ADO	0	0	-
BNAG	0	1 (0.87)	*p* = 1.000
DMAL	40 (88.88)	107 (93.85)	*p* = 0.322
LIP	0	0	-
IARL	0	0	-
DGLU	45 (100)	114 (100)	-
DMNE	45 (100)	114 (100)	-
TYRA	43 (95.55)	111 (97.36)	*p* = 0.622
CIT	0	0	-
NAGA	0	0	-
IHISA	0	0	-
ELLM	41 (91.11)	113 (99.12)	***p*** **= 0.023**
DCEL	0	0	-
GGT	0	0	-
BXYL	0	0	-
URE	0	1	*p* = 1.000
DTAG	19 (42.22)	53 (46.49)	*p* = 0.626
AGLU	0	0	-
ODC	44 (97.77)	109 (95.61)	*p* = 1.000
GGAA	0	3 (2.63)	*p* = 0.559
PYRA	0	0	-
AGLTP	0	0	-
DMAN	45 (100)	114 (100)	-
PLE	0	0	-
DTRE	45 (100)	114 (100)	-
SUCT	27 (60)	93 (81.57)	***p*** **= 0.004**
LDC	43 (95.55)	105 (92.10)	*p* = 0.730
IMLTA	1 (2.22)	5 (4.38)	*p* = 1.000
MNT	0	0	-
AGAL	42 (93.33)	109 (95.61)	*p* = 0.688
CMT	45 (100)	114 (100)	-
ILATA	2 (1.75)	9 (7.89)	*p* = 0.730
BGAL	45 (100)	113 (99.12)	*p* = 1.000
OFF	45 (100)	113 (99.12)	*p* = 1.000
BAIAP	0	0	-
DSOR	45 (100)	112 (98.24)	*p* = 1.000
5KG	0	1 (0.87)	*p* = 1.000
PHOS	7 (15.55)	35 (30.70)	*p* = 0.051
BGUR	23 (51.11)	38 (33.33)	***p*** **= 0.038**

APPA—Ala-Phe-Pro-arylamidase, H2S—H2S production, BGLU—Beta-glucosidase, PROA—L-Proline Arylamidase, SAC—Saccharose/sucrose, ILATK—L-lactate alkalinization, GLYA—Glycine arylamidase, O129R—O/129 resistance (comp.vibrio.), ADO—Adonitol, BNAG—Beta-N-acetyl-glucosaminidase, DMAL—D-maltose, LIP—Lipase, IARL—L-arbitol, DGLU—D-glucose, DMNE—D-mannose, TYRA—Tyrosine arylamidase, CIT—Citrate, NAGA—Beta-N-acetyl-galactosaminidase, IHISA—L-histidine assimilation, ELLM—Ellman, DCEL—D-cellobiose, GGT—Gamma-glutamyl-transferase, BXYL—Beta-xylosidase, URE—Urease, DTAG—D-tagatose, AGLU—Alpha-glucosidase, ODC—Ornithine decarboxylase, GGAA—Glu-Gly-Arg-arylamidase, PYRA—L-pyrrolydonyl-arylamidase, AGLTP—Glutamyl arylamidase pNA, DMAN—D-mannitol, PLE—Palatinose, DTRE—D-trehalose, SUCT—Succinate alkalinization, LDC—Lysine decarboxylase, IMLTA—L-malate assimilation, MNT—Malonate, AGAL—Alpha-galactosidase, CMT—Coumarate, ILATA—L-lactate assimilation, BGAL—Beta-galactosidase, OFF—Fermentation/glucose, BAIAP—Beta-alanine-arylamidase pNA, DSOR—D-sorbitol, 5KG—5-keto-D-gluconate, PHOS—Phosphatase, BGUR—Beta-glucuronidase. Statistical significance, *p* < 0.05 (bold).

**Table A4:** Detection of serum-susceptible (SS) and serum-resistant (SR) *E. coli* O25 ST131 isolates.

Isolate No.	Inactivated Serum (CFU)	Effect of Serum (CFU)	Colonies Killed by Serum (%)	SS/SR Isolate
1.	652	554	15	SR
2.	667	0	100	SS
3.	613	510	16	SR
4.	634	0	100	SS
5.	662	0	100	SS
6.	671	120	82	SR
7.	514	0	100	SS
8.	587	247	58	SR
9.	616	105	83	SR
10.	631	454	28	SR
11.	596	458	23	SR
12.	645	13	98	SS
13.	636	12	98	SS
14.	606	212	65	SR
15.	671	34	95	SS
16.	679	251	63	SR
17.	610	37	94	SS
18.	685	0	100	SS
19.	633	493	22	SR
20.	625	1	99.8	SS
21.	689	62	91	SS
22.	631	560	11	SR
23.	676	0	100	SS
24.	571	0	100	SS
25.	608	24	96	SS
26.	638	83	87	SR
27.	625	1	99.8	SS
28.	687	14	98	SS
29.	641	186	81	SR
30.	632	1	99.8	SS
31.	665	0	100	SS
32.	658	559	15	SR
33.	646	7	99	SS
34.	687	8	99	SS
35.	680	469	31	SR
36.	652	1	99.8	SS
37.	619	3	99.5	SS
38.	631	1	99.8	SS
39.	642	36	94	SS
40.	611	549	10	SR
41.	682	0	100	SS
42.	646	1	99.8	SS
43.	624	437	30	SR
44.	624	0	100	SS
45.	697	592	15	SR
46.	645	96	85	SR
47.	642	36	94	SS
48.	611	68	89	SR
49.	682	0	100	SS
50.	679	4	99.5	SS
51.	670	321	52	SR
52.	636	0	100	SS
53.	697	2	99.7	SS
54.	691	0	100	SS
55.	632	5	99.2	SS
56.	656	586	11	SR
57.	697	35	95	SS
58.	646	84	87	SR
59.	644	0	100	SS
60.	679	4	99.5	SS
61.	669	182	73	SR
62.	691	0	100	SS
63.	696	132	81	SR
64.	647	0	100	SS
65.	692	186	73	SR
66.	697	35	95	SS
67.	644	86	87	SR
68.	646	310	52	SR
69.	671	376	44	SR
70.	665	182	73	SR
71.	680	0	100	SS
72.	696	132	81	SR
73.	643	4	99.4	SS
74.	692	179	74	SR
75.	672	114	83	SR
76.	615	43	93	SS
77.	662	424	36	SR
78.	674	34	95	SS
79.	611	342	44	SR
80.	645	43	93	SS
81.	670	436	35	SR
82.	707	243	66	SR
83.	643	51	92	SS
84.	693	636	8	SR
85.	698	314	55	SR
86.	625	281	55	SR
87.	668	13	98	SS
88.	674	81	88	SR
89.	614	13	98	SS
90.	670	435	35	SR
91.	615	0	100	SS
92.	617	505	18	SR
93.	689	248	64	SR
94.	625	173	72	SR
95.	693	602	13	SR
96.	597	468	22	SR
97.	713	509	29	SR
98.	636	0	100	SS
99.	675	634	6	SR
100.	624	144	77	SR
101.	617	505	18	SR
102.	689	255	63	SR
103.	631	568	10	SR
104.	693	605	13	SR
105.	561	82	85	SR
106.	557	380	32	SR
107.	636	0	100	SS
108.	610	518	85	SR
109.	607	303	50	SR
110.	630	542	14	SR
111.	712	579	19	SR
112.	631	567	10	SR
113.	695	77	89	SR
114.	659	298	55	SR
115.	635	246	61	SR
116.	630	19	97	SS
117.	683	147	78	SR
118.	638	185	71	SR
119.	594	6	99	SS
120.	683	7	99	SS
121.	709	10	99	SS
122.	610	33	95	SS
123.	693	0	100	SS
124.	715	643	10	SR
125.	682	150	78	SR
126.	678	231	66	SR
127.	647	97	85	SR
128.	687	0	100	SS
129.	615	68	89	SR
130.	625	56	91	SS
131.	592	3	99.5	SS
132.	698	49	93	SS
133.	614	2	99.7	SS
134.	617	0	100	SS
135.	689	656	5	SR
136.	659	303	54	SR
137.	645	32	95	SS
138.	662	7	99	SS
139.	620	75	88	SR
140.	672	7	99	SS
141.	566	256	55	SR
142.	613	230	62	SR
143.	646	162	75	SR
144.	656	33	95	SS
145.	685	0	100	SS
146.	698	342	51	SR
147.	662	503	24	SR
148.	678	562	17	SR
149.	596	3	99.5	SS
150.	659	409	38	SR
151.	606	341	44	SR
152.	587	13	98	SS
153.	670	429	36	SR
154.	643	476	26	SR
155.	542	360	34	SR
156.	617	111	82	SR
157.	655	367	44	SR
158.	634	205	68	SR
159.	640	143	78	SR

CFU—colony-forming units, SR—serum-resistant, SS—serum-susceptible isolate.
